# Public Health Response to Multistate *Salmonella* Typhimurium Outbreak Associated with Prepackaged Chicken Salad, United States, 2018

**DOI:** 10.3201/eid2806.211633

**Published:** 2022-06

**Authors:** Bradford Greening, Hilary K. Whitham, Wade K. Aldous, Nancy Hall, Ann Garvey, Steven Mandernach, Emily B. Kahn, Patrick Nonnenmacher, Jason Snow, Martin I. Meltzer, Sandra Hoffmann

**Affiliations:** Centers for Disease Control and Prevention, Atlanta, Georgia, USA (B. Greening, Jr., H.K. Whitham, E.B. Kahn, P. Nonnenmacher, J. Snow, M.I. Meltzer);; State Hygienic Laboratory at the University of Iowa, Coralville, Iowa, USA (W.K. Aldous, N. Hall);; Iowa Department of Public Health, Des Moines, Iowa, USA (A. Garvey);; Iowa Department of Inspections and Appeals, Des Moines (S. Mandernach);; US Department of Agriculture, Washington, DC, USA (S. Hoffmann)

**Keywords:** *Salmonella*, *Salmonella enterica* serovar Typhimurium, food safety, public health economics, quantitative evaluation, health impact assessment, foodborne illness, enteric infections, bacteria, United States

## Abstract

Quantifying the effect of public health actions on population health is essential when justifying sustained public health investment. Using modeling, we conservatively estimated that rapid response to a multistate foodborne outbreak of *Salmonella* Typhimurium in the United States in 2018 potentially averted 94 reported cases and $633,181 in medical costs and productivity losses.

The US Centers for Disease Control and Prevention estimates that 48 million illnesses, 128,000 hospitalizations, and 3,000 deaths are caused by foodborne illnesses each year in the United States (*1*). *Salmonella* alone accounts for 1.35 million illnesses, 26,600 hospitalizations, and 421 deaths in the United States annually (*2*). Although incidence of *Salmonella enterica* serotype Typhimurium has declined since 2000, infection with this serotype continues to pose a public health burden because it can result in higher rates of hospitalization and longer lengths of stay in a hospital relative to other serotypes (*3*–*6*). A subset of *Salmonella* illnesses are identified and reported as part of an outbreak (defined as >2 persons who become ill from the same exposure); 96% of *Salmonella* outbreaks are caused by foodborne transmission (*7*). Outbreaks provide an opportunity to identify implicated food vehicles, as well as root causes for contamination, which can in turn inform broader food safety prevention efforts. If a *Salmonella* outbreak is suspected, public health officials can limit further cases by quickly identifying the source and issuing a recall for the implicated product or making other recommendations for restricting exposure to it.

## The Study

On February 5, 2018, staff at the State Hygienic Laboratory (SHL) at the University of Iowa (Coralville, Iowa) observed a notable increase in the number of stool samples submitted for *Salmonella* testing. Whole-genome sequencing and serotyping revealed patterns of genetic similarity between isolates obtained from these samples. By completing epidemiologic interviews of affected persons and performing food sample testing on suspected food products, the Foodborne Rapid Response team of the Iowa Department of Public Health (IDPH) was able to identify the source of the outbreak as prepackaged chicken salad sold by a Midwest grocery store chain. By February 9, the grocery store chain voluntarily removed the product from all of its Iowa stores; on February 13, IDPH and the Iowa Department of Inspections and Appeals (DIA) issued a joint consumer advisory notification warning customers that the product was implicated in multiple cases of *Salmonella* illness.

Using the PulseNet national molecular subtyping network for foodborne illness surveillance (a national laboratory network that compares the DNA of bacteria from patient samples to find clusters of disease that might represent unrecognized outbreaks), we identified a total of 265 persons from 8 states with *Salmonella* Typhimurium illness as part of this outbreak. Of those, 240 were from Iowa. Ninety-four hospitalizations were reported (35.5% of cases), including 1 person from Iowa who died. Decisive, cooperative actions undertaken between the Iowa SHL and IDPH, together with the Food and Consumer Safety Bureau within the Iowa DIA, resulted in the removal of contaminated product within 3 days from the initial identification of the genetically related samples, averting what could have been a larger outbreak.

Using a method reported by Scharff et al. (*8*), we estimated the number of cases of *Salmonella* Typhimurium averted by responding rapidly to this outbreak (Table 1). As a result of the alert raised by Iowa public health staff, the US Department of Agriculture (USDA) issued a recall for ≈20,630 pounds of potentially contaminated chicken salad from grocery stores in 8 states (*9*). According to USDA records, the manufacturer reported that 5,397 pounds (26.2%) of the product were recovered (*9*). Using estimates for product loss at the consumer level (*10*,*11*), we calculated that 13,481 pounds of the product were consumed, implying ≈20 confirmed cases of *Salmonella* Typhimurium per 1,000 pounds of product consumed. Assuming this rate of disease transmission also applied to the quantity of product that was successfully recalled, we conclude that ≈106 (99–114) cases of *Salmonella* Typhimurium were averted through the expedient recall of the chicken salad (i.e., cases that would have been reported had the recalled product been consumed). This estimate also assumes that all reported cases of *Salmonella* infection in the outbreak resulted from consumption of the contaminated product (Appendix 1)

**Table 1:** Quantitative analysis of cases averted in a multistate *Salmonella enterica* serovar Typhimurium outbreak, United States, 2018

Parameter	Value	Source
Product included in recall, lb	20,630	(*9*)
Product marked as recovered, lb (%)	5,397 (26.2)	(9)
Product available for consumption, lb	15,233	Calculated
Available product consumed, %	88.5 (82.0–94.5)	(*10,11*)
Product consumed, lb	13,481 (12,491–14,395)	Calculated
Outbreak cases reported	265	(*9*)
Cases/1,000 lb consumed	19.66 (18.41–21.22)	Calculated
Cases averted	94	Calculated
Cases averted including underdiagnosis	2,751	Calculated (*7*)
Hospitalizations averted	33	Calculated

*Values are no. (range) except as indicated.

Our estimate does not account for potential underdiagnosis resulting from variations in medical care seeking, specimen submission, and laboratory testing. Scallan et al. (*7*) estimated that, for every reported case of nontyphoidal *Salmonella*, 29.3 (90% credible interval 21.8–38.4) cases are likely not reported; therefore, the number of cases averted due to the product recall, including underdiagnosed cases, is estimated as 2,751 (range 2,047–3,605). Those cases would have occurred had the recalled product been consumed; of those, 94 would have been reported and the rest underdiagnosed. Our results assume the middle estimate for the fraction of available product consumed (Appendix 1 Table 4).

Using cost of illness estimates for nontyphoidal *Salmonella* generated by USDA/ERS (*12*), we estimated the economic costs to society averted by responding rapidly to this outbreak (Table 2). All costs are inflation-adjusted to 2018 US dollars. For our estimate of 94 cases averted, we calculated averted economic costs to society of $601,563 in direct medical costs and $31,618 in productivity losses resulting from missed working days in nonfatal cases. The total estimate of averted costs rises to $844,000 to $1 million when accounting for underdiagnosis. These numbers likely constitute an underestimate because we were conservative in selecting input parameters in cases where uncertainty or feasible ranges exist (Appendix 1). Furthermore, our analysis does not consider secondary effects that could provide additional benefits, such as prevention of future potential outbreaks through providing industry with information by which to improve their processes.

**Table 2:** Estimated economic impact of cases averted in a multistate *Salmonella enterica* serovar Typhimurium outbreak, United States, 2018*

Characteristic in underdiagnosis scenario	None	Low	Middle	High
Averted cases				
Underdiagnosis correction factor	0	21.8	29.3	38.4
No. cases averted	94	2,047	2,751	3,605
Economic impact of cases averted				
Medical costs, USD	$601,563	$673,626	$699,610	$731,137
Productivity loss, nonfatal cases				
Total lost working days	112.1	619.5	802.5	1,024.5
Total economic loss, USD	$31,618	$170,901	$221,123	$282,059
Total cost of illness, USD	$633,181	$844,526	$920,733	$1,013,196

*Data are for base case (middle) scenario for Fraction of Available Product Consumed. Appendix 1 Tables 5, 6 show results using sensitivity analysis scenarios. USD, US dollars.

## Conclusions

Quantifying and communicating effects such as the amount of illness and economic costs prevented by response and prevention efforts to policymakers and other appropriate audiences using a clear and systematic approach helps to show the value in investing in a robust, responsive, and collaborative public health infrastructure. Although data from outbreak events may lack some of the information desired for a direct calculation of the effect of interventions on population health, methods do exist that aid in making conservative estimates. Routinely calculating and communicating these estimates using direct and relatable outcome indicators for a variety of public health actions helps demonstrate the importance of investing in the ability to respond to outbreaks when they occur and of sustained investment in measures that prevent these outbreaks from occurring. Future analyses could expand upon our approach by examining the sequence of public health actions in relationship to the rise and fall of daily case counts, which may provide additional useful insights into the value of timely information. Incorporating information about the distribution of product sales and recovery could yield specific knowledge for future studies.

Appendix 1 Detailed methodology and results of sensitivity analyses in study of a multistate *Salmonella* Typhimurium outbreak associated with prepackaged chicken salad, United States, 2018.

Appendix 2 Data from analysis of public health response to multistate *Salmonella* Typhimurium outbreak associated with prepackaged chicken salad, United States, 2018.

Appendix 3 A portion of the success story written about the public health response to multistate *Salmonella* Typhimurium outbreak associated with prepackaged chicken salad, United States, 2018.
