# Supplementary material for: Public Health Response to Multistate Salmonella Typhimurium Outbreak Associated with Prepackaged Chicken Salad, United States, 2018
Source: Emerg Infect Dis. 2022 Jun;28(6):1254–6. doi: 10.3201/eid2806.211633 (PMC9155865; doi:10.3201/eid2806.211633)
Supplement: Appendix 1 — Detailed methodology and results of sensitivity analyses in study of a multistate Salmonella Typhimurium outbreak associated with prepackaged chicken salad, United States, 2018. [file 21-1633-Techapp-s1.pdf]

Article DOI: https://doi.org/10.3201/eid2806.211633

Public Health Response to Multistate *Salmonella* Typhimurium Outbreak Associated with Prepackaged Chicken Salad, United States, 2018

Appendix 1

Cases Averted

We used the product recall method described in Scharff et al. (*5*) to calculate the number of cases averted through the recall of the potentially contaminated chicken salad product. In short, this method calculates the rate of infection from the amount of product that is expected to have been consumed and applies that rate of infection to the amount of product that was successfully recovered by the recall to estimate how many cases may have been averted.

Following this method, we used the total amount of product in the batch of chicken salad that was designated for recall as well as the amount of product marked recovered by the manufacturer to calculate the amount of product that was still available for consumption. We reduced the estimate of product still available for consumption using a product consumption factor of 88.5%, i.e., that 88.5% of the product available for consumption was actually eaten by end consumers. We then calculated the rate of infection per 1,000 lbs. of product consumed and applied this rate of infection to the amount of product that was recovered to give us an estimate of the cases averted.

A search of the available data regarding food loss (that is, what quantities of various purchased food products are actually eaten by end consumers versus wasted) did not provide parameter values that were directly applicable to chicken salad. Therefore, we chose values from categories that were most closely aligned and performed a sensitivity analysis by varying the fraction of available product consumed from the base case value of 88.5% to a high scenario of 94.5% and a low scenario of 82.0% (Appendix 1 Table 2). These values are consistent with estimates of:

- Product losses of prepared foods by supermarkets (11.5% food loss = 88.5% of product consumed) (*3*)
- Product losses of deli products by supermarkets (5.5% food loss = 94.5% of product consumed) (*3*)
- Product losses of raw poultry by consumers (18.0% food loss = 82.0% of product consumed) (*2*)

Note that as the assumed fraction of available product consumed decreases (while known cases remains fixed), the cases per 1,000 lbs. of product consumed will increase (i.e., there would be a higher burden of illness per 1,000 lbs. of product consumed). The estimated number of cases averted does not change when varying the value for the fraction of available product consumed. This is because we had to assume that the rate of illness per 1,000 lbs. of product consumed for the recovered product would have been the same as the rate as calculated from the reported cases.

Underdiagnosis of Cases

Underdiagnosis of *Salmonella* Typhimurium and other foodborne illness can occur due to variations in medical care seeking, specimen submission, laboratory testing, and test sensitivity. We accounted for these variations by using underdiagnosis multipliers that were obtained from a study by Scallan et al. (*4*) Note that this study assumes that there is no underreporting for salmonellosis, but only underdiagnosis. We used the multiplier value given (29.3) and performed sensitivity analysis by using the low and high endpoints of the range provided (21.8, 38.4). This allowed us to provide a range of cases potentially averted by accounting for cases that may not have sought care (Appendix 1 Table 3). A recently released study by Collier et al. (*6*) provides a similar estimated underdiagnosis multiplier of 29.1. Note that both of these underdiagnosis multipliers were estimated using national-level data from the United States and thus may have differed from the actual underdiagnosis that occurred in the specific jurisdictions affected by this outbreak.

Cost of Illness Estimates

As the basis for our cost analysis, we used a standardized cost-of-illness tool for non-typhoidal *Salmonella* that was implemented in Microsoft Excel by USDA/ERS (available at https://www.ers.usda.gov/webdocs/DataFiles/48464/Salmonella_2018.xlsx?v = 7698.4). This

tool contains national-level cost per case estimates for the United States given in 2018 US dollars. Methodological details for the estimates and calculations provided in the cost-of-illness tool can be found at https://www.ers.usda.gov/data-products/cost-estimates-of-foodborne-illnesses/documentation. For convenience, we have included the ‘per case assumptions’ section of this tool in our own Excel calculator tool that contains all calculations used in this analysis (Appendix 2, https://wwwnc.cdc.gov/EID/article/28/6/21-1633-App2.xls). We now describe how the cost-per-case estimates were incorporated into this analysis.

This tool divides cases into 4 categories:

1. Non-hospitalized, did not visit physician, recovered
2. Non-hospitalized, visited outpatient physician, recovered
3. Hospitalized, with post-hospitalization recovery
4. Hospitalized, died

We will now give some details on how we divided case estimates among these categories. (Methodological details on how these categorizations were converted into cost estimates can be found at the documentation link for the original ERS calculator, listed immediately above.) Steps taken and assumptions made to produce the results shown here can be found in the Excel tool provided (Appendix 2).

We began by calculating a hospitalization rate of 35.5% based on data in the ELC outbreak report / success story write-up (Appendix 3, https://wwwnc.cdc.gov/EID/article/28/6/21-1633-App3.pdf) that 94 of the 265 cases were hospitalized. The case report further noted that 1 hospitalized patient died, a death rate among hospitalized patients of 1.06% (1/94). This left 171 non-hospitalized patients, to which we applied a “physician visit rate” of 7.33%, which we derived from national case burden estimates provided in the USDA/ERS calculator tool (on the “Per Case Assumptions” tab). These same methods and derived values were applied to the case totals that were adjusted for underdiagnosis (both reported cases and averted cases), with the only additional detail being that we assumed that none of the underdiagnosed cases (those in excess of the 265 reported or 94 calculated averted cases) were hospitalized.

Summary tables were created using appropriate totals from the calculator tool (Appendix 2). Note that we did not include estimated costs of premature death in our results, since all scenarios resulted in less than 1 averted death. However, in other analyses assessing the impact of a public health response to outbreaks, it may be appropriate to include estimates of the cost of premature death. For such analyses, the ability to estimate the cost of premature death due to nontyphoidal *Salmonella* is provided by the USDA/ERS calculator tool, and questions regarding valuing mortality outcomes can be directed to Sandra Hoffmann (shoffmann@ers.usda.gov).

Caveats and Limitations

The USDA/ERS cost of illness calculator used was originally designed using nationally representative costing estimates for the U.S. for non-typhoidal *Salmonella*. Cases of *Salmonella* Typhimurium can be more serious, with both higher hospitalization rates and longer durations in hospital, on average, relative to other serotypes (*7–9*). We calculated the hospitalization rate using data taken directly from the outbreak report, but data regarding length of hospital stay for individual cases from this outbreak were not available. Additionally, despite studies indicating that *Salmonella* Typhimurium can have longer hospital stays relative to other serotypes, data were not available that allowed us to calculate how this translates to an increase in direct medical costs. The nationally representative cost estimates from the USDA/ERS calculator tool were used, with the understanding that they may be a slight underestimate of the treatment costs incurred in this outbreak. Combining all these points, we believe that the results presented here constitute a conservative cost estimate and have made every effort to list the data used as well as limitations where necessary.

Sensitivity Analysis Results

There were two inputs to this analysis for which parameter values were notably uncertain: 1) the amount of underdiagnosis of salmonellosis cases and 2) the fraction of the affected food product that was actually consumed. We performed scenario-based sensitivity analysis across a chosen range of values for these two parameters, producing results using a high, medium, and low value for each parameter. However, the estimated number of cases does not change when varying the value for the fraction of available product consumed (see above for explanation). Therefore, we present only the results from varying the underdiagnosis correction factor. Table 2 in the main text shows the estimates for cases and costs averted due to the

expedient recall of the contaminated product, while Appendix 1 Table 4 below provides estimates of economic impact for cases that were reported.

There were 265 cases reported across multiple states as having been related to this contaminated food source (chicken salad). When accounting for expected underdiagnosis, we estimate that the true number of cases likely lies between 5,777–10,176 (Appendix 1 Table 4). This works out to an estimated economic impact of U.S.$1.90–2.06 million in direct medical costs and U.S.$2.38–$2.86 million when including productivity losses. This is a slight increase from the estimated economic impact without accounting for underdiagnosis, which is expected since the "missed" cases are, in general, not expected to have incurred very much in the way of direct medical costs, which comprise the bulk of the estimated economic impact.

References

1. US Department of Agriculture, Food Safety and Inspection Service. Recalls and public health alerts. 2019 May 13 [cited 2019 Dec 6]. https://www.fsis.usda.gov/wps/portal/fsis/topics/recalls-and-public-health-alerts/recall-case-archive/recall-case-archive-2018

2. Muth MK, Karns SA, Nielsen SJ, Buzby JC, Wells HF. Consumer-level food loss estimates and their use in the ERS loss-adjusted food availability data. Washington, D.C.: United States Department of Agriculture, Economic Research Service; 2011 Jan. technical bulletin no. 1927. Contract no. 59–4000–6-0121. https://www.ers.usda.gov/webdocs/publications/47570/8043_tb1927.pdf

3. Food Marketing Institute. Technomic Inc. The sophistication of supermarket fresh prepared foods (but not just the food). 2016. [cited 2019 Dec 6]. https://www.fmi.org/forms/store/ProductFormPublic/sophistication-of-supermarket-fresh-prepared-foods

4. Scallan E, Hoekstra RM, Angulo FJ, Tauxe RV, Widdowson MA, Roy SL, et al. Foodborne illness acquired in the United States—major pathogens. Emerg Infect Dis. 2011;17:7–15. PubMed https://doi.org/10.3201/eid1701.P11101

5. Scharff RL, Besser J, Sharp DJ, Jones TF, Peter GS, Hedberg CW. An economic evaluation of PulseNet: a network for foodborne disease surveillance. Am J Prev Med. 2016;50(Suppl 1):S66–73. PubMed https://doi.org/10.1016/j.amepre.2015.09.018

6. Collier SA, Deng L, Adam EA, Benedict KM, Beshearse EM, Blackstock AJ, et al. Estimate of burden and direct healthcare cost of infectious waterborne disease in the United States. Emerg Infect Dis. 2021;27:140–9. PubMed https://doi.org/10.3201/eid2701.190676

7. Kennedy M, Villar R, Vugia DJ, Rabatsky-Ehr T, Farley MM, Pass M, et al.; Emerging Infections Program FoodNet Working Group. Hospitalizations and deaths due to *Salmonella* infections, FoodNet, 1996–1999. Clin Infect Dis. 2004;38(Suppl 3):S142–8. PubMed https://doi.org/10.1086/381580

8. Santos AC, Roberts JA, Cook AJ, Simons R, Sheehan R, Lane C, et al. *Salmonella* Typhimurium and *Salmonella* Enteritidis in England: costs to patients, their families, and primary and community health services of the NHS. Epidemiol Infect. 2011;139:742–53. PubMed https://doi.org/10.1017/S0950268810001615

9. Chen Y, Glass K, Liu B, Hope K, Kirk M. *Salmonella* infection in middle-aged and older adults: incidence and risk factors from the 45 and up study. Foodborne Pathog Dis. 2016;13:689–94. PubMed https://doi.org/10.1089/fpd.2016.2170

Appendix 1 Table 1. Parameters and values used in study of public health response to an outbreak of *Salmonella* Typhimurium from prepackaged chicken salad, 2018

Parameter	Value (sensitivity analysis values)	Source
Qty of product recalled, lbs	20,630	(*1*)
Qty of product marked as recovered, lbs	5,397	(*1*)
Fraction of available product consumed	88.5% (82.0%, 94.5%)	(*2*,*3*)
Underdiagnosis multiplier	29.3 (21.8, 38.4)	(*4*)
Total reported cases	265	Appendix 3
Hospitalizations	94	Appendix 3
Hospitalized, died	1	Appendix 3
Hospitalization rate, %	35.5	Calculated
% Hospitalized that died	1.06	Calculated
% Nonhospitalized that visited physician (outpatient visit)	7.33	*Assumption*
% Hospitalized in underdiagnosed population only	0	*Assumption*

Appendix 1 Table 2. Fraction of product consumed used for sensitivity analysis of an outbreak of *Salmonella* Typhimurium from prepackaged chicken salad, 2018

% of available product consumed	Cases averted	Source
94.5	94	(*3*)
88.5	94	(*3*)
82.0	94	(*2*)

Appendix 1 Table 3. Estimate of cases averted accounting for underdiagnosis of *Salmonella* Typhimurium from prepackaged chicken salad, 2018*

Underdiagnosis multiplier	Cases Averted
21.8	2,047
29.3	2,751
38.4	3,605

*Adjustment for under-diagnosis due to variations in medical care seeking, specimen submission, laboratory testing, and test sensitivity. Source: https://www.cdc.gov/eid/article/17/1/p1-1101-techapp3.pdf.

Appendix 1 Table 4. Estimated economic impact of reported cases in outbreak of *Salmonella* Typhimurium from prepackaged chicken salad, 2018

Description	Estimated economic impact			
Reported cases				
Underdiagnosis correction scenario	None	Low	Mid	High
Underdiagnosis correction factor	0	21.8	29.3	38.4
# of cases reported	265	5,777	7,765	10,176
	Economic impact (reported cases)			
Underdiagnosis correction scenario	None	Low	Mid	High
Medical costs	$1,697,907	$1,901,304	$1,974,645	$2,063,631
Productivity loss, nonfatal cases				
Total lost working days	316.5	1,748.6	2,265.0	2,891.6
Total economic loss ($US)	$89,242	$482,366	$624,118	$796,110
Total cost of illness	$1,787,149	$2,383,670	$2,598,762	$2,859,741
