# Supplementary material for: Public Health Response to Multistate Salmonella Typhimurium Outbreak Associated with Prepackaged Chicken Salad, United States, 2018
Source: Emerg Infect Dis. 2022 Jun;28(6):1254–6. doi: 10.3201/eid2806.211633 (PMC9155865; doi:10.3201/eid2806.211633)
Supplement: Appendix 3 — A portion of the success story written about the public health response to multistate Salmonella Typhimurium outbreak associated with prepackaged chicken salad, United States, 2018. [file 21-1633-Techapp-s3.pdf]

Article DOI: https://doi.org/10.3201/eid2806.211633

Public Health Response to Multistate *Salmonella* Typhimurium Outbreak Associated with Prepackaged Chicken Salad, United States, 2018

Appendix 3

ELC Outbreak Report/Success Story

The analysis presented in this article was based on a success story submitted to CDC's Epidemiology and Laboratory Capacity for Prevention and Control of Emerging Infectious Diseases (ELC) Cooperative Agreement. For over 25 years, the ELC Cooperative Agreement has provided funding to US public health entities at the state, local, territorial, and other administrative levels to detect, respond to, control, and prevent outbreaks of infectious disease. We include a portion of this success story write-up to provide first-hand details not otherwise included in the main text.

Iowa Foodborne Rapid Response Team Quickly Identifies Chicken Salad as the Source of a Multistate *Salmonella* Typhimurium Outbreak

Problem or Challenge

When two or more people become ill after consuming the same food or drink, the event is considered a foodborne disease outbreak. CDC estimates 48 million people get sick from foodborne illnesses, 128,000 are hospitalized and 3,000 die from foodborne diseases each year. If an outbreak is suspected, public health officials need to quickly identify the source to control the spread of the outbreak and reduce the number of affected individuals. While most outbreaks tend to be locally confined and affect maybe only a few people, regional or national outbreaks may affect hundreds of people. This story is about a regional multistate foodborne outbreak that was first detected in Iowa.

The state of Iowa has a foodborne rapid response team (RRT) ready to take immediate action whenever there is a suspected foodborne outbreak. This multifunctional team comprises laboratory staff from the State Hygienic Lab that performs testing of both clinical and food/environmental specimens, epidemiologists from the Iowa Department of Public Health to investigate acute outbreaks, and technical staff of the Food and Consumer Safety Bureau within the Iowa Department of Inspection and Appeals that conduct food safety inspections at food establishments and processing plants and collects environmental and/or food samples for lab testing. In Iowa, the ELC program provides salary support for both IDPH epidemiology and SHL laboratory staff as well as funding for various laboratory testing areas for surveillance and disease detection.

Program Activity/Description/Strategy

During the first week of February, SHL lab staff observed a dramatic increase in the number of stool samples submitted for *Salmonella* culture. Most of these grew *Salmonella* Typhimurium isolates, which were characterized by pulsed-field gel electrophoresis (PFGE) and whole genome sequencing (WGS) as part of the CDC's PulseNet system and found to have similar patterns. IDPH epidemiologists interviewed affected individuals to identify potential food products and point sources causing the outbreak. Staff from the Iowa Department of Inspections and Appeals collected food and environmental samples from suspected sources for testing and comparison with clinical samples. Food sample testing was performed by SHL's environmental microbiology staff (Appendix 3 Figures 1 and 2). Whole-genome sequencing (WGS) was completed locally at the state level, after which CDC staff analyzed the data to show relatedness between isolates.

Within 3 days, the Iowa foodborne rapid response team was able to link the source of the outbreak to prepackaged chicken salad sold by a Midwest grocery store chain, which promptly removed all remaining product and notified consumers. Further environmental testing identified the production source of the chicken salad and a product recall was announced. A joint announcement by the Iowa Department of Public Health (IDPH) and the Iowa Department of Inspections and Appeals (DIA) was released on February 13, 2018. Steve Mandernach, Bureau Chief for Food and Consumer Safety of the Iowa Department of Inspection and Appeals praised SHL's laboratory testing efforts to quickly identify the source of the outbreak. The State

Hygienic Laboratory later developed a one-page handout to share with laboratory facilities during the joint 2018 American Society for Clinical Laboratory Science (ASCLS-IA) and Clinical Laboratory Management Association (CLMA) Iowa Meeting in April 2018.

This became a multistate outbreak when additional cases were identified via the PulseNet system from other states where the grocery store chain is located. CDC collaborated with public health and regulatory officials in several states and the U.S. Department of Agriculture's Food Safety and Inspection Service.

Outcomes/Impact/Value

The PulseNet system is a national laboratory network that connects foodborne illness cases to detect outbreaks across the country. When the PulseNet system was first established, collaborating laboratories used pulsed-field gel electrophoresis (PFGE) technology to create a DNA fingerprint of the tested organism which could then be compared to fingerprints submitted by other laboratories across the country to detect thousands of local and multistate outbreaks. The PulseNet system is currently migrating to a newer technology, whole genome sequencing, to detect and solve outbreaks faster with greater accuracy. During this outbreak, the State Hygienic Laboratory used both PFGE and DNA sequence analysis to rapidly confirm the source and reduce the spread of disease along with their partners on the Iowa foodborne rapid response team. SHL staff are truly dedicated to providing the best possible service to the citizens of Iowa. They are helping to reduce the spread of disease and performing a valuable public health mission. In this example, most of the cases were limited within the state of Iowa even though the affected grocery chain has locations in several Midwestern states. The food product was quickly removed from the shelves averting what could potentially have been a much larger outbreak. The outbreak received a lot of television, radio, and press coverage within the state as well as regionally.

Abbreviated CDC Outbreak Summary

Epidemiologic and laboratory evidence implicated chicken salad produced by an Iowa food processing plant and sold exclusively by a Midwest grocery store chain as the likely source of this multistate outbreak. While public health officials in Iowa first detected this outbreak, the CDC searched the PulseNet database and identified a total of 265 people from 8 states with *Salmonella* Typhimurium illness as part of this outbreak (Appendix 3 Table). The majority of

affected individuals (240) were from Iowa. Ninety-four hospitalizations were reported, including one person from Iowa who died.

Appendix 3 Table. Number of ill persons by state of residence in study of multistate Salmonella Typhimurium outbreak, 2018.

State	Case count
Illinois	10
Indiana	1
Iowa	240
Minnesota	4
Mississippi	1
Nebraska	5
South Dakota	3
Wisconsin	1
Total	265

Source: CDC. Multistate outbreak of *Salmonella* Typhimurium linked to chicken salad (final update). 2018 Apr 16 [cited 2018 Jul 20]. https://www.cdc.gov/salmonella/typhimurium-02-18/index.html

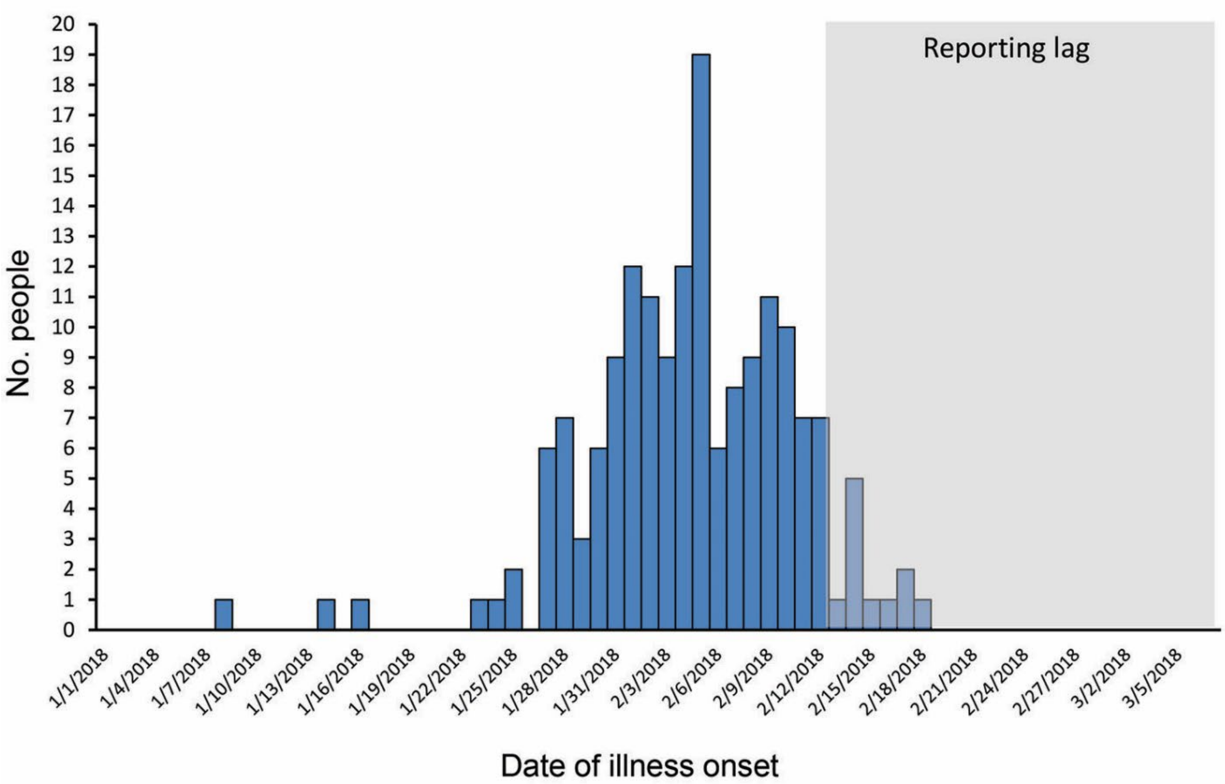

Appendix 3 Figure 1. Persons infected with the outbreak strains of *Salmonella* Typhimurium, by date of illness onset. Information was reported for 265 persons as of April 4, 2018. Some illness onset dates have been estimated from other reported information.

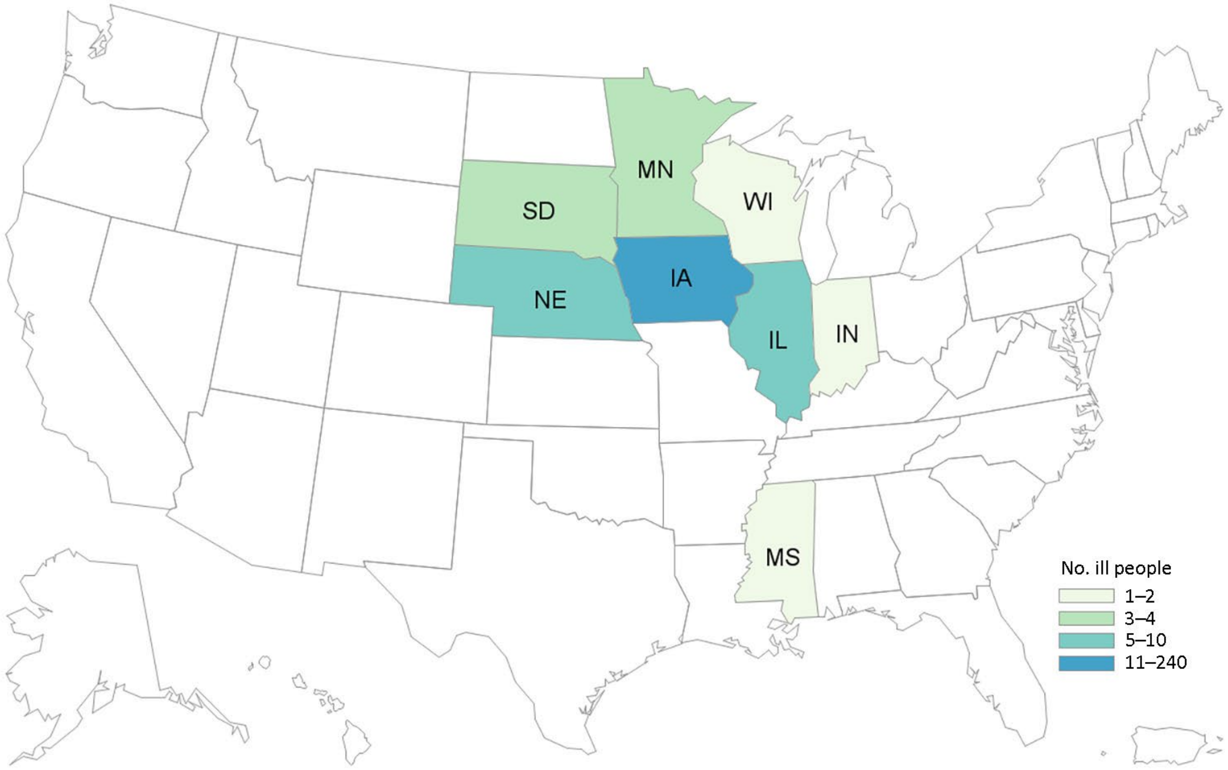

Appendix 3 Figure 2. Persons infected with the outbreak strains of *Salmonella* Typhimurium, by state of residence, as of April 4, 2018 (n = 265).
